# Lactate Dehydrogenase as a Biomarker in Oral Submucous Fibrosis: A Systematic Review and Meta-Analysis

**DOI:** 10.7759/cureus.51008

**Published:** 2023-12-23

**Authors:** Monalisha Mahapatra, Abikshyeet Panda, Harish Kumar, Diplina Barman, Rounik Talukdar, Prachurya Dakshinakabat

**Affiliations:** 1 Oral and Maxillofacial Pathology, Kalinga Institute of Dental Sciences, Bhubaneswar, IND; 2 Oral Pathology, Kalinga Institute of Dental Sciences, Bhubaneswar, IND; 3 Department of Public Health Dentistry, Indian Council of Medical Research (ICMR) National Institute of Cholera and Enteric Diseases (NICED), Kolkata, IND; 4 Department of Community Medicine, Indian Council of Medical Research (ICMR) National Institute of Cholera and Enteric Diseases (NICED), Kolkata, IND; 5 Oral pathology, Kalinga Institute of Dental Sciences, Bhubaneswar, IND

**Keywords:** oral squamous cell carcinoma, systematic review, oral submucous fibrosis, lactate dehydrogenase, biomarker

## Abstract

This systematic review and meta-analysis was planned with the objective of evaluating the level of Lactate Dehydrogenase (LDH) in oral submucous fibrosis patients and in controls and comparing them. For this meta-analysis, we searched Google Scholar, PubMed, Scopus, and Directory of Open Access Journals (DOAJ) databases using a search methodology that included combinations of MeSH terms and keywords and included cross-sectional studies to evaluate the levels of LDH in patients with Oral Submucous Fibrosis (OSMF), Oral Squamous Cell Carcinoma (OSCC) and compared it with the controls. The total number of records identified through database searching was 4161 (n). Analysis of the quality of the studies was done using the National Heart, Lungs and Blood Institute (NHLBI) tool for case-control studies. Twelve case-control studies which matched the inclusion criteria were included after the literature search. The meta-analysis was carried out using R Studio (version 4.1.3, 2022; The R Foundation for Statistical Computing, Vienna, Austria). The pooled estimate that has been calculated from the salivary LDH course for OSMF was 15.35% and from the serum LDH course for OSMF was 6.82%. There was a visual observation of the funnel’s plot asymmetry suggesting publication bias. After adjusting the publication bias, the t^2^ values for salivary and serum LDH were 41% and 14.71%, respectively, which was less than 50%, indicating that the meta-analysis was statistically significant. The evaluation of salivary and serum LDH can be a useful method for early diagnosis of OSMF as well as OSCC. To infer that individuals may have OPMD or OSCC, specific values for salivary and serum LDH must be found in further investigations.

## Introduction and background

Oral Squamous Cell Carcinoma (OSCC) is the sixth most prevalent malignant epithelial neoplasm of the oral cavity worldwide [1]. There are various etiological and predisposing factors for OSCC, which include alcohol, tobacco smoking, UV radiation, viral infections, hereditary genetic abnormalities, and immunological disorders [2]. OSCC development is a multistep process, its main precursors are Oral Potentially Malignant Disorders (OPMD) [3]. Native to the Indian subcontinent, Oral Submucous Fibrosis (OSMF) has a malignant potential of 4.5% to 7.6% among all OPMDs [4-6]. OSMF can develop at any age, although teenagers and adults are the most frequently affected and the important factor is known to be the consumption of areca nuts [7,8].

Early OPMD identification and therapy can stop the condition from progressing to OSCC. In the early diagnosis and detection of OPMD as well as OSCC, biochemical markers are crucial diagnostic tools. In the saliva, serum, and tissue of individuals with various diseases, we may identify several biochemical markers. [5] One of these markers is Lactate Dehydrogenase (LDH) which is widely distributed in the serum and saliva of patients with various OPMDs. When a cell dies, the components present in the cytoplasm, such as LDH, get released into the cytoplasm. It is thought that elevated serum lactate dehydrogenase (LDH) activity is a sign of cellular necrosis. Higher levels of LDH are a result of higher mitotic activity and increased synthesis of lactic acid by the tumor cells due to the breakdown of glycoproteins [6].

Various pathologies such as abdominal and lung cancer, teratoma, toxic hepatitis, megaloblastic anemia, progressive muscular dystrophy, myocardial infarction, and pulmonary embolism also result in higher levels of serum LDH [9]. Otto Warburg noted in 1927 that malignant cells metabolically depend primarily on enhanced glycolysis preceded by fermentation of lactic acid in the presence of oxygen. Metabolic remodeling of malignant cells alters metabolic pathways, reorganizing the Krebs cycle and promoting glycolysis.

They also stated that a significant quantity of lactate released by tumor cells into the extracellular environment lowers its pH to 6.0-6.5, promoting angiogenesis which serves as a metabolic fuel for tumor cells and suppresses the immune system [5]. It is believed that this tissue, rather than the various salivary glands, is the source of LDH in saliva since its characteristics mimic those of the epithelial tissue of the mouth [10,11]. Since LDH is a biological expression of necrosis, any circumstance that affects the oral epithelium may result in a change in the amount of LDH in saliva. This implies that LDH concentration in saliva may be used as a specific signal for oral lesions that affect the integrity of the mucosal surface [12]. Similarly, a rise in blood LDH levels indicates that there is more frequent necrotic activity inside the tumor cell. In this systematic review and meta-analysis, we evaluated as well as compared the level of salivary and serum LDH in OSMF to that of healthy individuals.

## Review

Materials and methods

This systematic review was planned with the objective of evaluating the level of LDH in oral submucous fibrosis patients and in healthy individuals an comparing them. The systematic review has been registered in the Open Science Framework (OSF) with registration DOI 10.17605/OSF.IO/CZWN7.

Focused Question

The following focused question was constructed: Can the estimation of lactate dehydrogenase level in oral submucous fibrosis patients be used as a biomarker?

Selection Criteria

All the articles were evaluated and criteria for inclusion and exclusion were set (Table 1).

**Table 1:** Inclusion and exclusion criteria for the selected studies

Inclusion Criteria	Exclusion Criteria
Original studies, such as longitudinal or case-control studies, published in indexed scientific journals were included.	Studies not reporting the value of salivary LDH were excluded.
Studies including patients diagnosed both clinically and histopathologically of oral submucous fibrosis and compared with healthy individuals.	We excluded studies that did not mention oral submucous fibrosis in particular and those including adolescents under 18 years of age.
Studies that have free full text available and that have been written in the English language are included.	Review articles, experimental studies, case reports, and letters to the editor are excluded.

Search Strategies

The search was conducted from 15th June 2022 till 1st July 2022. The databases that were searched include Google Scholar, PubMed, Web of Science, Scopus, and Directory of Open Access Journals (DOAJ). We kept our initial search as broad as possible. The search strategy included a combination of various keywords such as lactate dehydrogenase, biomarker, oral potentially malignant disorders, premalignant lesions, premalignant conditions, and oral submucous fibrosis. The search strategies included for PubMed, Google Scholar, and Scopus are mentioned in Table 2. We searched the keywords, keeping it all in the title. 

**Table 2:** Search theme for the systematic review

Databases	Search theme
Central search theme (PubMed)	((lactate dehydrogenase) OR (salivary lactate dehydrogenase)) OR (lactate dehydrogenase enzyme)) OR (lactate dehydrogenase biomarker)) AND (oral precancer)) OR (pre-malignant disorder)) OR (premalignant lesion)) OR (oral potentially malignant disorder)) OR (potentially malignant disorder)) OR (oral submucous fibrosis)) OR (submucous fibrosis)).
Search theme for Google Scholar	
Search theme for Scopus	(lactate dehydrogenase) OR (salivary lactate dehydrogenase)) OR (biomarker)) AND (oral submucous fibrosis)) (lactate dehydrogenase) OR (salivary lactate dehydrogenase)) OR (lactate dehydrogenase enzyme)) OR (lactate dehydrogenase biomarker)) AND (oral submucous fibrosis)) OR (submucous fibrosis) (lactate dehydrogenase) OR (salivary lactate dehydrogenase)) OR (biomarker)) AND (oral submucous fibrosis))

Expected outcomes

In this systematic review, we checked the lactate dehydrogenase levels in patients with OSMF and controls and compared them. We expected our study to give a positive comparative value so that LDH can be proved to be an important biomarker for oral submucous fibrosis and can be helpful in early diagnosis, in therapeutics as well as in finding treatment outcomes.

Data Extraction

Researchers checked search outcomes to make sure they were comprehensive after finishing the study selection, and repetitions were then eliminated. The complete title and abstract of the articles were individually checked by the reviewers. All qualifying articles were checked by the first and second author for eligibility using the following standards: first author, publication year, region of origin, study title, study type, the area from where the study groups were recruited, inclusion and exclusion criteria, characteristics of the population, type of saliva and serum collection, processing and measuring procedures. Salivary and serum LDH levels were evaluated in all groups (control group, OSCC, OSMF, oral leukoplakia (OL), tobacco chewers) by the third and sixth authors. The statistical significance of each study was evaluated by the fourth and fifth authors.

Quality Assessment

The methodological quality of the selected studies was evaluated using the National Heart, Lungs and Blood Institute (NHLBI) tool for case-control studies. The scores awarded were 1 for yes, 2 for no, 3 for not applicable/not clear. The included experiments were marked as good, fair, and poor.

Data Synthesis and Analysis

The main outcome of this study was a positive comparative value so that LDH can be proved to be an important biomarker for oral submucous fibrosis. The meta-analysis was carried out using R Studio (version 4.1.3, 2022; The R Foundation for Statistical Computing, Vienna, Austria). We utilized the specialized command package 'metaprop' (Rdocumentation.org) for meta-analysis of single proportions. The standard error (SE) of the percentage of individuals who used dental services according to the study's established criteria was calculated using the information that was obtained [13]. The following is a representation of the formula: SE=√(P×(1-P) ∕ n), P=proportions, n=population size [14].

Proportions were pooled using the random effect inverse variance approach, which requires determining a weighted average using SE and sending it to the data frame for the meta prop package in the R studio. The combined result was presented as a percentage with a 95% confidence interval, and a forest plot was created if needed for graphical presentation. I^2^ statistics were used to determine the degree of heterogeneity. The I^2^ values for heterogeneity assessment were in the following categories: mild (25%), moderate (between 25 and 75%), and extreme (>75%) [15]. On the basis of the year of the research, the levels of serum and salivary LDH, a subgroup assessment was carried out to assess heterogeneity. To identify the likely cause of heterogeneity for the subsequent variables: year of research, concentrations of serum and salivary LDH in patients with OSMF, concentrations of serum and salivary LDH in controls, univariate meta-regression was used. A funnel plot was used to assess publication bias, and Egger's test was used to determine if the plot's asymmetry was present. A p-value of 0.05 or below was regarded as statistically significant [16].

To further assess the accuracy of our estimate, trim and fill analyses were carried out by computing the modified pooled prevalence [17]. The test accomplishes this by attempting to balance the funnel plot by balancing the pooled prevalence by recalculating it while taking into account any potential missing studies as a result of publication bias [18]. For the evaluation, the built-in "trimfill.rma.uni" function from the "metafor" R package was used. Furthermore, a funnel plot was made with inputs for unavailable studies and an updated utilization estimate [19].

Results

Study Selection

**Figure 1:**
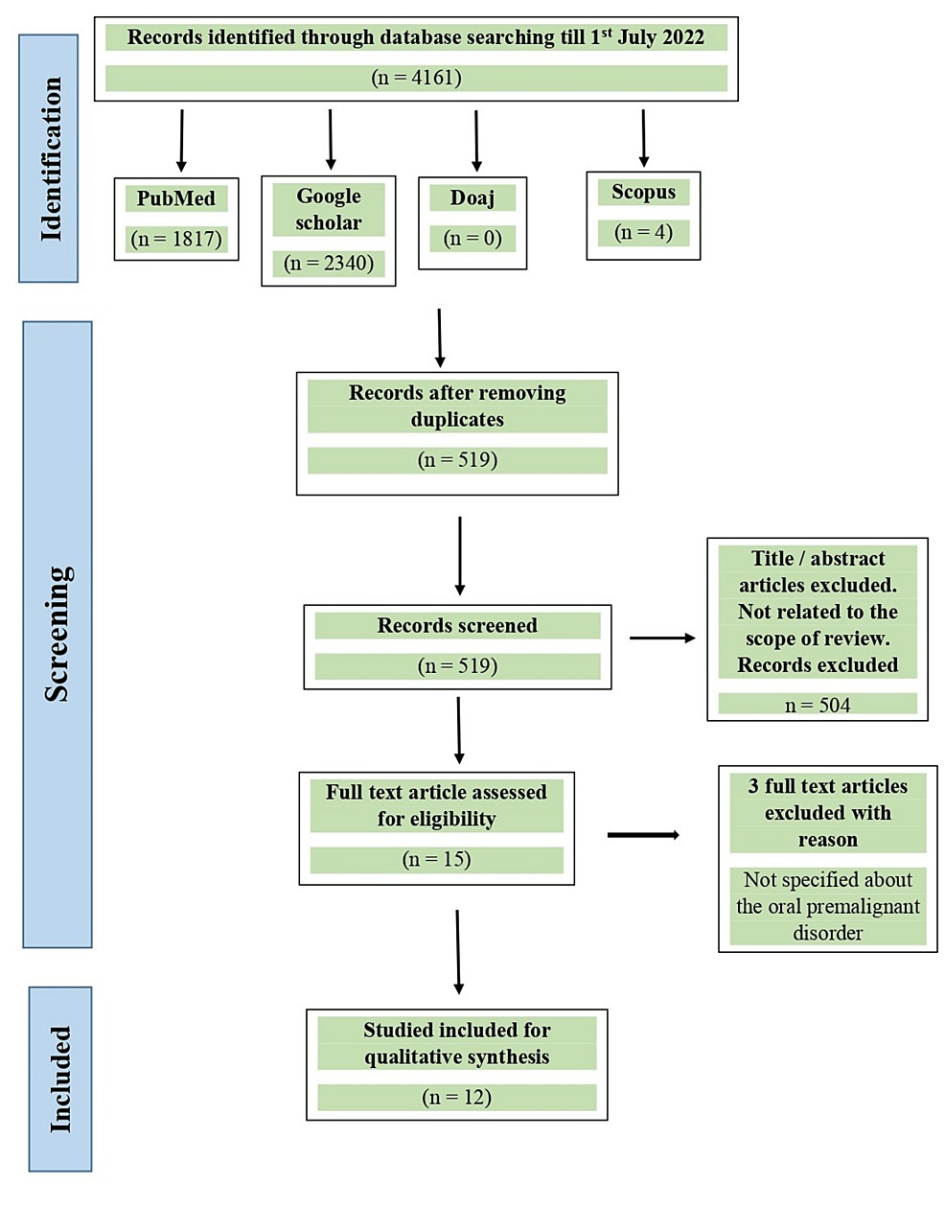
Illustration of search results according to PRISMA guidelines PRISMA: Preferred Reporting Items for Systematic Reviews and Meta-Analyses.

A total of 4161 articles were found in PubMed, Google Scholar, Scopus and DOAJ. After eliminating duplicates, 519 of the 4161 results that the selection method produced remained. According to the criteria for inclusion and exclusion, all titles were examined to produce 15 articles and 504 articles were excluded as they were not related to the scope of review. After carefully going through the 15 complete texts, we eliminated the three articles (n=3) that did not specifically address any oral potentially malignant disorder. [1,5,10,20-31].Finally, we included 12 articles in the systematic review as they matched the eligibility criteria (Figure 1).

Study Characteristics

We selected 12 studies that matched our inclusion and exclusion criteria. Out of these, 11 studies were case-control studies, one of which was a prospective comparative study. These studies were published between 2015 and 2020. Five hundred and sixty patients in all were enrolled, of which 300 had OSMF, 90 had oral leukoplakia, 140 had OSCC, 30 had smoked, and 285 had been in good health. one study compared OSMF with healthy controls [20]. Five out of 12 studies evaluated both salivary and serum LDH levels [1,20,23,24,28]. Two selected studies [6,25] mentioned the study duration. All the studies evaluated oral submucous fibrosis out of which three studies [23,25,26] compared OSMF with OL, two studies [23,27] compared OSMF with OSCC and OL, three studies [10,28,30] compared OSMF with OSCC and one study [1] compared OSMF with OSCC and individuals with tobacco chewing habits.

All of the chosen studies had eligibility and exclusion criteria. Four out of 12 studies evaluated the salivary LDH level [10,22,25,31] and three out of 12 studies [26,27,30] evaluated the serum LDH level. All the studies collected the unstimulated whole saliva except one [20] collected the stimulated whole saliva. One out of 12 studies [25] used the UV semi-automated spectrophotometer method, two studies [20,24] used the LDH assessment kit [24], five studies used [1,10,22,28,31] semi-auto analyzer, and two studies [23,25] used spectrophotometry (340 nm) for analysis [25]. The most common method used was a semiauto analyzer. Three [1,23,27] out of 12 studies collected serum samples from the peripheral veins. Four [6,20,26,28] out of 12 studies collected the serum samples by venipuncture. One out of 12 studies [24] used vacutainer needles for serum sample collection. Three [6,27,28] out of 12 studies used semi-automatic analyzers for serum LDH measurement. Three [1,20,24] out of 12 studies used an LDH assessment kit for serum LDH measurement. Two [23,26] out of 12 studies used spectrophotometers for serum LDH measurement.

Data Extraction

Data extraction of each article was done through an Excel sheet. Separate columns were made for each variable that we selected. Variables that were included are study type, publication year, sample sizes of diseased and control groups, age group, average ages of diseased and healthy individuals, study duration, number of males and females, LDH level in saliva and serum of diseased, i.e., OSMF, OSCC, oral leukoplakia and in healthy individuals, i.e., control, salivary and serum LDH in patients with habit, their p values, primary outcomes and major themes (Table 3). We did the quality evaluation of all studies according to the NHLBI guidelines [29].

**Table 3:** Findings from the reviewed studies NA: Not applicable.

SL NO.	AUTHOR YEAR	AGE GROUP	Salivary LDH control (IU/L)	Salivary LDH OSMF (IU/L)	Salivary LDH OSCC (IU/L)	Serum LDH control (IU/L)	Serum LDH OSMF (IU/L)	Serum LDH OSCC (IU/L)
1	Shah et al, 2017 [5]	18–60	159.2504	299.86	331.1538	303.3222	403.1696	398
2	Samlin et al, 2019 [10]	40-65	398.104	747.1	1172.5	NA	NA	NA
3	Sivaramakrishnan et al, 2015 [20]	18–60	80.73	606.83	NA	289.4	521	NA
4	Kallalli et al, 2016 [22]	20–70	182.21	608.28	630.96	NA	NA	NA
5	Gantala et al, 2018 [23]	25-75	200.3	490.5	1023	221.6	540.6	1076
6	Mishra et al, 2018 [24]	18–60	668.25	1057.3	NA	313.05	408.35	NA
7	Mantri et al, 2019 [25]	18–70	86.12	350.43	592.09	NA	NA	NA
8	Anthwal et al, 2020 [26]	22-55	NA	NA	NA	160.49	241.4	NA
9	Rathore et al, 2015 [27]	20-60	NA	NA	NA	161.39	249.68	323.83
10	Panda et al, 2020 [28]	20–70	140.62	631.67	NA	217.09	534.58	NA
11	Swamy et al, 2016 [30]	20-50	NA	NA	NA	117.23	492.2	NA
12	Kadiyala et al, 2015 [31]	30-50	126.7	612.2	515.7	NA	NA	NA

Main Findings

In all of the examined studies, OSCC and OPMD categories had higher serum and salivary concentrations of LDH than the healthy individuals' group [5,10,20,22-27,28,30,31]. Three eligible studies [10,23,28] evaluated higher LDH levels in the OL group when compared to the control group but lower when compared to OSCC. In all of the included trials, the concentration of serum and salivary LDH were higher in OSMF cases in comparison to the controls [5,10,12,20,22-28,30]. All the studies showed statistically significant “p” value. 

Quality Assessment

The National Heart, Lung, and Blood Institute (NHLBI) tool was used to assess the quality of evidence obtained from the chosen studies. The complete tool website is https://www.nhlbi.nih.gov/health-topics/study-quality-assessment-tools. As the studies included in this study were case-control studies we choose NHLBI criteria for case control studies for scoring which included 12 questions (Table 4). The scores awarded were 1 for yes, 2 for no, and 3 for not applicable/ not clear. There were 12 questions that were answered individually for all studies and was rated as good, fair and poor quality following the investigators decision. We decided to mark 60-70% as good, above 50% as fair, and below 50% as poor for a particular study. According to this we found two of our studies to be good, eight of our studies to be fair and two of our studies to be of poor quality (Table 4).

**Table 4:** Quality assessment of the included studies Includes the quality assessment done for various studies that are included in the systematic review. SL NO refers to the question number that is mentioned above. The scores awarded were 1 for yes, 2 for no, and 3 for not applicable/not clear.

SL NO.	Shah et al. [5]	Samlin et al. [10]	Sivarama krishnan et al. [20]	Kallalli et al. [22]	Gantala et al. [23]	Mishra et al. [24]	Mantri et al. [25]	Anthwal et al. [26]	Rathore et al. [27]	Panda et al. [28]	Swamy et al. [30]	kadiyala et al. [31]
Was the research question or objective in this paper clearly stated and appropriate?	1	1	1	1	1	1	1	1	1	1	1	2
Was the study population clearly specified and defined?	1	1	1	1	1	1	1	1	1	1	1	1
Did the authors include a sample size justification?	2	2	2	2	2	2	2	2	2	2	2	2
Were controls selected or recruited from the same or similar population that gave rise to the cases (including the same timeframe)?	1	1	1	1	1	1	1	1	2	1	1	1
Were the definitions, inclusion and exclusion criteria, algorithms or processes used to identify or select cases and controls valid, reliable, and implemented consistently across all study participants?	1	1	1	1	1	1	1	1	1	1	1	1
Were the cases clearly defined and differentiated from controls?	1	1	1	1	1	1	1	1	1	1	2	1
If less than 100 percent of eligible cases and/or controls were selected for the study, were the cases and/or controls randomly selected from those eligible?	3	3	3	3	3	3	3	3	3	3	3	3
Was there use of concurrent controls?	2	2	2	2	2	2	2	2	2	2	2	2
Were the investigators able to confirm that the exposure/risk occurred prior to the development of the condition or event that defined a participant as a case?	2	2	1	2	2	1	1	2	2	2	2	2
Were the measures of exposure/risk clearly defined, valid, reliable, and implemented consistently (including the same time period) across all study participants?	2	2	2	2	2	1	1	2	2	2	2	2
Were the assessors of exposure/risk blinded to the case or control status of participants?	2	2	2	2	2	2	2	2	2	2	2	2
Were key potential confounding variables measured and adjusted statistically in the analyses? If matching was used, did the investigators account for matching during the study analysis?	1	1	1	1	1	1	1	1	1	1	1	1

Meta-Analysis

An illustration of a forest plot often serves as the primary output of a meta-analysis. Effect size is represented on the X-axis, which is located at the top. With the exception of the bottom row, each row displays the study's effect size in points and its 95% confidence interval. The region where the "true" effect will be found is shown by the 95% CI. Here, we shall evaluate the measured impact magnitude with the findings from various investigations. In a meta-analysis, we assure that every study included is a study of a complete probability sample of the desired population.

**Figure 2:**
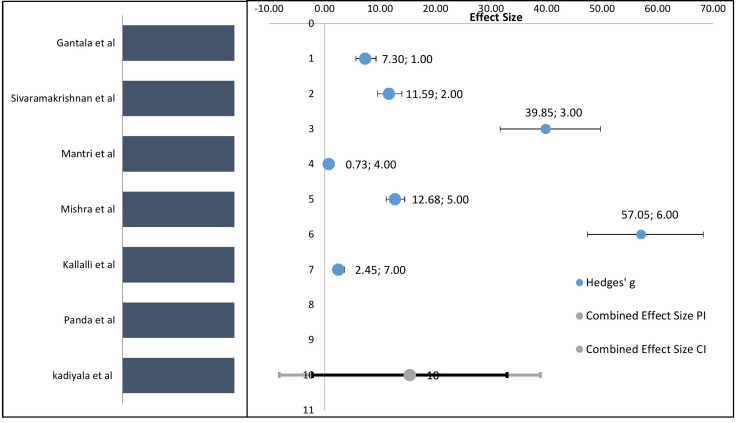
Forest plot showing variation of the salivary LDH scores for OSMF when compared to control LDH levels OSMF: Oral Submucous Fibrosis; LDH: Lactate Dehydrogenase.

If we see the studies, we included the effect size of salivary LDH estimation by Gantala et al. [23] was 7.30 (CI: 5.64-9.25%), Sivaramakrishnan et al. [20] was 11.59 (CI: 9.57-13.92%), Mantri et al. [25] was 39.85 (CI: 31.62-49.70%), Mishra et al. [24] was 0.73 (CI: 0.05-1.44%), Kallalli et al. [22] was 12.68 (CI: 11.10-14.42%), Panda et al. [28] was 57.05 (CI: 47.36-68.24%), and Kadiyala SV [31] was 2.45 (CI: 1.51-3.53%). Majority of the studies have been reported between the effect size within the lower range of 7.30 and the upper range of 57.05 (Figure 2). The pooled estimate that has been calculated from the salivary LDH course for OSMF was 15.35%. The pooled estimate is the weighted average effect.

**Figure 3:**
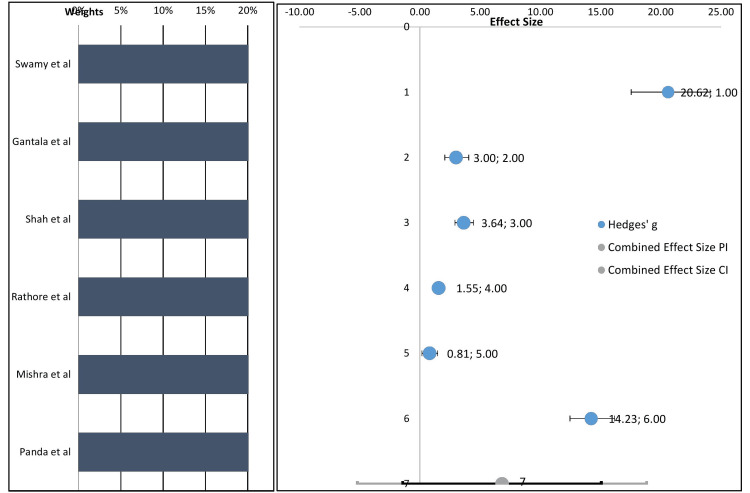
Forest plot showing the variation of the serum LDH scores for OSMF when compared to control LDH levels OSMF: Oral Submucous Fibrosis; LDH: Lactate Dehydrogenase.

If we see the studies, we included the effect size of serum LDH estimation by Swamy and Ganiger [30] was 20.62 (CI: -11.88 to -7.37%), Gantala et al. [23] was 3.00 (CI: 2.14 to 4.15%), Shah et al. [5] was 3.64 (CI: -2.88 to -1.58%), Rathore et al. [27] was 1.55 (CI: -1.03 to 0.34%), Mishra et al. [24] was 0.81 (CI: -1.78 to -0.95%), Panda et al. [28] was 14.23 (CI: -44.13 to -30.61%). The majority of the studies have reported the effect size within the lower range of 0.81 and the upper range of 20.62. The pooled estimate that has been calculated from the serum LDH course for OSMF was 6.82% (Figure 3). The pooled estimate is the weighted average effect. In the future, if any one conducts a study their values should fall under this 95% prediction level.

Risk of Bias

**Figure 4:**
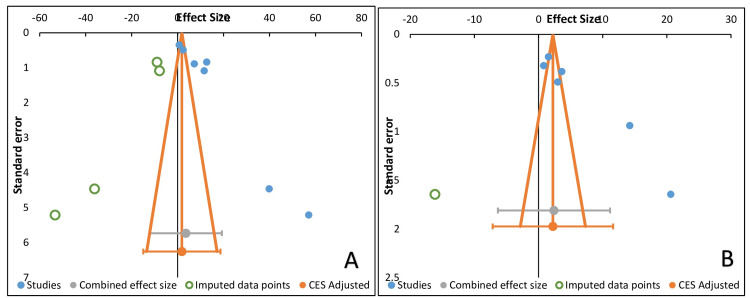
Publication bias study results Trim and fill analysis for mentioning publication bias (A and B).

There was visual observation of funnel plot asymmetry so to assess Egger’s test. It was conducted to check for potential publication bias in the literature in the present meta-analysis. The structure of Egger's test funnel plot showed that it was indicative of publication bias. The Duval and Tweedie "trim and fill" approach was employed to account for publication bias while assessing the quantity of LDH in saliva and serum of OSMF patients. After adjusting the publication bias, t^2^ values for salivary and serum LDH were 41% and 14.71%, respectively, which was less than 50% indicating that the meta-analysis was statistically significant (Figure 4).

Discussion

Early oral submucous fibrosis detection may benefit from the use of biomarkers as a tool for diagnosis during dental screenings [20,21]. The presence of tumor markers in serum, tissue, and saliva can aid in the identification of neoplastic processes [23,24,10]. Serum has traditionally been the preferred medium for the detection of biochemical markers, although it has certain limitations. Instead, saliva acts as a non-invasive, widely accessible diagnostic medium for identifying OSMF. One such biomarker that we discussed here is LDH. LDH is a physiological enzyme that is found in the cytoplasm of all healthy live cells and becomes extracellular during the death of cells. In anaerobic glycolysis, LDH acts as an intracellular enzyme and catalyzes the process of lactate synthesis through pyruvate degradation [25,26].

OSMF is a chronic condition of the mouth that is characterized by a sub-epithelial inflammatory response that leads to fibroelastic alterations in the submucosa [27,23]. Hypoxia, changes in glycolysis, and fibrosis are potential contributors to the LDH enzyme activity in OSMF. In order to find out the levels of LDH in OSMF patients and to compare them with healthy individuals we conducted this systematic review and meta-analysis by detailed literature search.

This is the first systematic review and meta-analysis that examines whether blood and salivary LDH levels are greater in OSMF than in healthy persons. In this systematic review, we discovered that OSMF and OSCC have greater amounts of LDH than the controls.

We found out that amounts of LDH can be a useful tool for assessing the effectiveness of treatment for OSMF which was supported by Sivaramakrishnan et al. (2015) [28]. According to some authors, the increasing amount of LDH could be linked to the Warburg effect which states that regardless of situations where there is oxygen, enhancing glycolysis is the primary source of energy for cancer cells, after which comes lactic acid fermentation. Upto forty times as much lactic acid as normal cells are thought to be produced by cancer cells. Thus, regardless of normoxic environments, the Warburg effect causes cancerous transformation of cells to result in increased glucose uptake and a larger proportion of pyruvate turning into lactate [5].

In our literature search, we found out that when compared to female respondents, male subject’s salivary LDH levels were generally greater [27]. Further, we saw that a significant connection exists between serum LDH levels and tumor differentiation. The amount of LDH in saliva can be utilized to replace invasive and expensive diagnostic techniques. A novel, reliable, and user-friendly technique for diagnosing oral potentially malignant and malignant disorders in their early stages is the salivary LDH estimate [10]. LDH in the serum and saliva may be quantified and used as a biomarker.

After conducting this systematic review we ruled out various criteria that would support the use of LDH as a biomarker. The release of enzymes into the bloodstream by cancerous tumor cells or nearby tissue destroyed by the tumor adds to an aberrant rise in enzyme levels [30]. Here, the result showed that the level of LDH is higher in OSMF than in healthy individuals and the level of LDH is higher in OSCC than in OSMF. As discussed above, an elevated mitotic index and higher lactic acid generation by tumor cells as a result of the disintegration of glycoprotein.

OSMF patients have higher blood LDH levels [32]. As a result, measuring the amount of LDH in patients with OSMF can aid in the early identification of these alterations and serve as a potential biomarker for screening OSCC and monitoring the progression of OPMD lesions. In accordance with our exclusion and inclusion criteria, this study has some shortcomings. Here, we included only those studies that were written in the English language but studies in other languages might be present. Secondly, we excluded the studies in which the values of LDH were not mentioned and particularly OSMF was not mentioned. The different sample sizes and research designs across the included studies also contribute to some methodological variability. Also, all the studies that were included by us were case-control studies involving a variety of restrictions, lastly, the other factors that affect the variations present among investigations could be the different methods for saliva and serum sampling used by every study and different methods used for LDH evaluation.

As we can see, all the studies included by us are conducted in India so we believe that studies should also be conducted in different parts of the country to verify the results obtained. Saliva pre-collection measures taken by each study were different, but in most studies, unstimulated whole saliva was used which was taken in the morning at the resting state, but in the study conducted by Sivaramakrishnan et al. (2015) stimulated whole saliva was used for evaluation. For the measurement of LDH, most of them used a semi-automatic analyzer, but some of them also used an LDH assessment kit and spectrophotometer for quantification. Similarly, for the collection of serum samples, in most of the studies, they collected the venous blood by vein puncture but some of them collected samples from the peripheral veins. For quantification of LDH, most of them used a semi-automatic analyzer but some of them also used an LDH assessment kit and spectrophotometer. It can be noticed that although various studies used similar techniques, their results were highly variable. This could be because the technique used in most research varies greatly and is not clearly stated. In different studies, different procedures of centrifugation, i.e., speed and duration were followed ranging from 4-15 minutes. In future studies, it is necessary to determine a particular value for salivary and serum LDH to conclude that may be having OPMD or OSCC.

Strength and limitations

A novel, reliable, and user-friendly technique for diagnosing oral potentially malignant and malignant disorders in their early stages is the salivary LDH estimate [10]. The amount of LDH in saliva and serum can be a useful tool for assessing the effectiveness of the treatment for OSMF. The non-invasive procedure of collection of saliva for quantification of LDH does not require a proper setup which may not be available at all times.

This systematic review has some shortcomings as we included only those studies that were written in the English language but studies in other languages might be present. Secondly, we excluded the studies in which the values of LDH were not mentioned and particularly OSMF was not mentioned. The different sample sizes and research designs across the included studies also contribute to some methodological variability. Also, all the studies that were included by us were case-control studies involving a variety of restrictions, lastly, the other factors that affect the variations present among investigations could be the different methods for saliva and serum sampling used by every study and different methods used for LDH evaluation. For the measurement of LDH, most of them used a semi-automatic analyzer but some of them also used an LDH assessment kit and spectrophotometer for quantification. Also, the sample pre-collection measures taken are different in various studies. All these methodological differences present in the studies can cause variations in the level of LDH.

## Conclusions

In this systematic review of the articles, we found that in healthy individuals, the LDH level in the saliva ranges from 80.73-668.25 IU/L, and in OSMF patients, it ranges from 299.86-1057.3 IU/L. Similarly, the LDH level in the serum of healthy individuals ranges from 117.23-313.05 IU/L and in OSMF patients, ranges from 241.4-540.6 IU/L, which suggests that levels of salivary and serum LDH of an individual beyond the range which is seen in controls are helpful in establishing the fact that increase amount of LDH suggests the presence of OSMF in the oral cavity which can be diagnosed on clinical examination so that early treatment can be done and further progression of the disease can be prevented. In our forest plot the pooled estimate for serum LDH course for OSMF was 6.82% and for salivary LDH course was 15.35%. This systematic review and meta-analysis study suggests that the amount of serum and salivary LDH is high in OSMF when compared with healthy individuals despite various methodological differences, and if any future studies are conducted, the values should fall under our 95% prediction level.
